# Association of markers of endothelial function with blood‐based biomarkers of Alzheimer’s Disease in individuals of admixed ancestry

**DOI:** 10.1002/alz.092401

**Published:** 2025-01-09

**Authors:** Nicholas R. Ray, Jiji T. Kurup, Kara L. Hamilton‐Nelson, Anthony J. Griswold, Brian W. Kunkle, William S. Bush, Giuseppe Tosto, Adesola Ogunniyi, Rufus Akinyemi, Jonathan L. Haines, Goldie S. Byrd, Jeffery M. Vance, Margaret A. Pericak‐Vance, Gary Beecham, Christiane Reitz

**Affiliations:** ^1^ Columbia University Irving Medical Center, New York, NY USA; ^2^ John P. Hussman Institute for Human Genomics, University of Miami Miller School of Medicine, Miami, FL USA; ^3^ John P. Hussman Institute for Human Genomics, University of Miami Miller School of Medicine, Miami, FL, USA, Miami, FL USA; ^4^ Department of Population and Quantitative Health Sciences, Institute for Computational Biology, Case Western Reserve University, Cleveland, OH USA; ^5^ G.H. Sergievsky Center, Vagelos College of Physicians and Surgeons, Columbia University, New York, NY USA; ^6^ University of Ibadan, Ibadan Nigeria; ^7^ Maya Angelou Center for Health Equity, Wake Forest University School of Medicine, Winston‐Salem, NC USA; ^8^ John P. Hussman Institute for Human Genomics, Miami, FL USA; ^9^ John P. Hussman Institute for Human Genomics, Miller School of Medicine, Miami, FL USA; ^10^ Dr. John T. Macdonald Foundation Department of Human Genetics, University of Miami Miller School of Medicine, Miami, FL USA; ^11^ G. H. Sergievsky Center, Vagelos College of Physicians and Surgeons, Columbia University, New York, NY USA

## Abstract

**Background:**

While Alzheimer disease (AD) is the most common cause of dementia, it has long been recognized that cardiovascular and cerebrovascular health plays a major role in cognitive function. As such, the development of accessible biomarkers to assess vascular cognitive impairment and dementia (VCID) is a key step towards identifying effective prevention and treatment strategies. While a set of blood‐based VCID biomarkers has been under investigation, there is a critical paucity of data from genetically admixed individuals. As such, the role and impact of genetic ancestry and the validity these biomarkers in diverse populations is unknown. To address this, we assessed the associations between biomarkers of endothelial function, AD, and genetic ancestry.

**Method:**

Biomarkers include amyloid beta (Aβ)42/40 and phosphorylated tau (p‐tau)/Aβ42 ratios for AD, and vascular endothelial growth factor (VEGF), placenta growth factor (PlGF), basic fibroblast growth factor (bFGF), vascular cell adhesion protein 1 (VCAM‐1), and intercellular adhesion molecule‐1 (ICAM‐1) for VCID. Biomarkers were collected from 256 admixed individuals sampled from Puerto Rico and Peru, and African Americans from the US. Including age and sex as covariates, mixed effects regressions were modeled separately predicting AD biomarker ratios by all VCID biomarkers, degree of African ancestry, and APOE genotype.

**Result:**

Aβ42/40 ratio was associated with VEGF (β = ‐1.97×10^‐5^, P = .002), bFGF (β = ‐2.10×10^‐4^, P = .02), VCAM‐1 (β = 1.53×10^‐5^, P = 9.02×10^‐4^), ICAM‐1 (β = ‐3.72×10^‐5^, P = .01) and degree of African ancestry (β = 0.01, P = .04). Similarly, p‐tau/Aβ42 ratio was associated with VEGF (β = 0.01, P = .001), bFGF (β = 0.18, P = 5.21 × 10^‐5^), VCAM (β = ‐0.005, P = .02), and ICAM (β = 0.02, P = .02). No significant associations were observed for PlGF.

**Conclusion:**

Blood‐based measures of endothelial function markers VEGF, bFGF, ICAM‐1, and VCAM‐1 are associated with both the p‐tau/Aβ42 and Aβ42/40 ratios, the latter of which is also modulated by degree of African ancestry. These observations support the notions that these CVD biomarkers may be valuable biomarkers for cognitive health in admixed individuals and inform the design of clinical trials for biomarker development across populations.

